# Future directions of Doctor of Public Health education in the United States: a qualitative study

**DOI:** 10.1186/s12889-021-11086-z

**Published:** 2021-06-03

**Authors:** Chulwoo Park, Gene Migliaccio, Mark Edberg, Seble Frehywot, Geralyn Johnson

**Affiliations:** 1 Department of Public Health and Recreation, San Jose State University, 1 Washington Sq, San Jose, CA 95192 USA; 2 Department of Global Health, The George Washington University Milken Institute School of Public Health, 950 New Hampshire Ave NW #2, Washington, D.C. USA; 3 Department of Prevention and Community Health, The George Washington University Milken Institute School of Public Health, 950 New Hampshire Ave NW #2, Washington, D.C. USA

**Keywords:** American Public Health Association (APHA), Association of Schools and Programs of Public Health (ASPPH), Council on Education for Public Health (CEPH), Doctor of Public Health (DrPH), Education, Public Health Professional, Master of Public Health (MPH), Qualitative Research

## Abstract

**Background:**

The Doctor of Public Health (DrPH) degree is an advanced and terminal professional degree that prepares the future workforce to engage in public health research, teaching, practice, and leadership. The purpose of the present research was to discuss the desirable future direction and optimal education strategies for the DrPH degree in the United States.

**Methods:**

A total of 28 Council on Education for Public Health (CEPH)-accredited DrPH programs in the United States was identified through the Association of Schools and Programs of Public Health (ASPPH) Academic Program Finder. Then, a qualitative analysis was conducted to obtain perspectives from a total of 20 DrPH program directors through in-depth interviews.

**Results:**

A DrPH program should be recognized as equal but different from an MPH or a PhD program and strengthen the curriculum of methodology and leadership education. It is important that a DrPH program establishes specific partnerships with other entities and provide funding for students. In addition, rather than being standardized nationwide, there is value in each DrPH program maintaining its unique character and enabling students to be open to all career pathways.

**Conclusions:**

The future of DrPH programs in the twenty-first century should aim at effective interdisciplinary public health approaches that draw from the best of both academic and applied sectors. A DrPH program is expected to provide academic, applied public health, and leadership training for students to pursue careers in either academia or the public/private sector, because public health is an applied social science that bridges the gap between research and practice.

**Supplementary Information:**

The online version contains supplementary material available at 10.1186/s12889-021-11086-z.

## Background

In the mid-to-late nineteenth century, most public health positions in the United States were part-time jobs with unstable incomes, lasting only for a political season, and therefore public health was not quite considered a “career” [1]. At the time, there was no standardized public health training system to educate future health officials; governors, mayors, or city councils appointed health officials through political patronage or bonds of friendship [1]. Since William Henry Welch and Wickliffe Rose outlined the template for public health professional education in the United States in 1915, efforts to develop and establish Doctor of Public Health (DrPH) education in the United States have been continuous [2]. At the 48th American Public Health Association (APHA) annual meeting in New Orleans in 1919, a committee consisting of 16 experts duly recognized the DrPH degree as a standardized professional training [3, 4]. In 1953, the Association of Schools of Public Health (ASPH) was established to enhance academic public health programs [5, 6]. In 1974, APHA and ASPH established the Council on Education for Public Health (CEPH), the independent agency that accredits schools of public health and public health programs [7, 8].

The original purpose of the DrPH program was to bridge the gap between research and applied fieldwork by training students to become cross-disciplinary researchers, researcher-practitioners, transdisciplinary scientists, and integration and implementation scientists. Rather than focusing on rigorous research skills, DrPH programs emphasize knowledge translation and transformative leadership [9]. A DrPH degree pursues interprofessional education, “a necessary step in preparing a collaborative, practice-ready health workforce that is better prepared to respond to local health needs,” defined by the World Health Organization [10]. In addition, the DrPH applied practice experience enables students to engage in collaborative practice with professionals outside of public health to satisfy interprofessional education requirements. DrPH programs have been encouraging DrPH students to become collaborative, practice-ready health workers, or researchers with strong leadership skills who understand how to effectively collaborate and work in an interprofessional environment.

However, little is known about how each of the CEPH-accredited schools and programs interpreted and provided practice- and leadership-oriented DrPH education. In fact, in contrast to the standardization effort in the 1900s, DrPH education in the twenty-first century in the United States has formed different program types: school/schoolwide (college/collegewide), school/departmental (college/departmental), and program/concentration. Depending on the program type, a core curriculum has been interpreted differently at each institution. For example, while the departmental type has provided five core public health specialties (e.g., epidemiology, biostatistics, health policy and management, environmental health, and social and behavioral sciences), the schoolwide (collegewide) and program/concentration types have provided unique concentrations (e.g., implementation science, health equity, social justice, preventive care, leadership in public health, and clinical laboratory science and practice).

Over the past years, very few studies have been conducted to comprehensively search the current list of CEPH-accredited DrPH programs and collect insights from experts about the future education and training required for a DrPH degree. While most studies regarding CEPH and a DrPH degree were quantitative, no clear evidence of qualitative data analyses has been reported. Qualitative research would uncover professional opinions and deep intuitive understanding of the future of DrPH education. Thus, the purpose of this research was to answer the following research question about doctoral education and public health through a qualitative study: What is the desirable future direction and optimal education strategy for a DrPH degree in the United States? This research has raised an important question about the necessity of standardization of DrPH education across the country for increasing its brand awareness and establishing a clear identity/purpose of a DrPH degree, under the circumstance that each DrPH program has its own trajectory.

## Methods

We reviewed the literature to have a better understanding of the existing analysis about DrPH education and training from particular programs or schools as well as from general perspectives in the United States [9, 11–20]. There were three criteria of identifying DrPH programs: 1) program locations: The United States and its territories, 2) program quality: CEPH-accredited, and 3) recruitment status: active for the 2021–2021 academic year. To identify the current CEPH-accredited DrPH programs, ASPPH’s Academic Program Finder was mainly used [21]. As of February 2020, the ASPPH’s Academic Program Finder listed a total of 30 CEPH-accredited DrPH programs. Of those 30 programs, we excluded 2 of them; the PhD in Nutritional Sciences at the University of Michigan School of Public Health was incorrectly included, and the DrPH program at the University of Pittsburgh Graduate School of Public Health was no longer accepting students for the 2020–2021 academic year. Thus, a total of 28 programs fit the criteria of CEPH-accredited DrPH programs that had a student recruitment plan for the 2020–2021 academic year. Then, we cross-checked those 28 programs searched from the ASPPH’s Academic Program Finder with the list of accredited DrPH schools or programs in the CEPH webpage [22]. We also conducted manual Google searching to identify additional DrPH programs accredited by CEPH. Manual Google searching identified six additional DrPH programs: East Carolina University, Florida A&M University Institute of Public Health, Indiana University Richard M. Fairbanks School of Public Health, Jackson State University School of Public Health, Morgan State University School of Community Health and Policy, and Ponce Health Sciences University. However, we ultimately did not include those additional DrPH programs in the final list because it was uncertain to know whether they received DrPH-specific CEPH accreditation, and besides, there might be a possibility of missing any other CEPH-accredited DrPH programs from manual Google searching. Thus, we decided to use the most conservative method, identifying DrPH programs that were searchable in the ASPPH’s Academic Program Finder. After taking all those situations into account, a total of 28 programs that met the criteria of CEPH-accredited DrPH programs were identified for inclusion in the present study (Table 1). The final list of programs included programs presented in Table 1 was based on conservative inclusion approach that a DrPH program should be clearly searchable both in the ASPPH’s Academic Program Finder and the CEPH webpage and recruited students in the 2020–2021 academic year.

**Table 1:** CEPH-accredited DrPH programs (This list is accurate as of February 2020) [23]

University or School Name (in alphabetical order)	Program Type	City, State
1. Boston University School of Public Health	School/Schoolwide	Boston, Massachusetts
2. Claremont Graduate University School of Community & Global Health	School/Schoolwide	Claremont, California
3. Colorado School of Public Health	School/Departmental	Aurora, Colorado
4. Columbia University Mailman School of Public Health	School/Departmental	New York, New York
5. Drexel University Dornsife School of Public Health	School/Departmental	Philadelphia, Pennsylvania
6. East Tennessee State University College of Public Health	School/Departmental	Johnson City, Tennessee
7. George Washington University Milken Institute School of Public Health	School/Departmental	Washington, DC
8. Georgia Southern University Jiann-Ping Hsu College of Public Health	School/Departmental	Statesboro, Georgia
9. Georgia State University School of Public Health	School/Schoolwide	Atlanta, Georgia
10. Harvard T. H. Chan School of Public Health	School/Schoolwide	Boston, Massachusetts
11. Johns Hopkins Bloomberg School of Public Health	School/Schoolwide	Baltimore, Maryland
12. Loma Linda University School of Public Health	School/Schoolwide	Loma Linda, California
13. New York Medical College School of Health Sciences	Program/Concentration	Valhalla, New York
14. Pennsylvania State University College of Medicine Public Health Program	Program/ Concentration	Hershey, Pennsylvania
15. SUNY Downstate Medical Center School of Public Health	School/Schoolwide	Brooklyn, New York
16Texas A&M School of Public Health	School/Schoolwide	College Station, Texas
17. Tulane University School of Public Health and Tropical Medicine	School/Departmental	New Orleans, Louisiana
18. University at Albany School of Public Health	School/Schoolwide	Rensselaer, New York
19. University of Alabama at Birmingham School of Public Health	School/Departmental	Birmingham, Alabama
20. University of Arizona Mel and Enid Zuckerman College of Public Health	College/Departmental	Tucson, Arizona
21. University of Arkansas Fay W. Boozman College of Public Health	College/Collegewide	Little Rock, Arkansas
22. University of California Berkeley School of Public Health	School/Schoolwide	Berkeley, California
23. University of Georgia College of Public Health	School/Departmental	Athens, Georgia
24. University of Illinois at Chicago School of Public Health	School/Schoolwide	Chicago, Illinois
25. University of North Carolina Gillings School of Global Public Health	School/Departmental	Chapel Hill, North Carolina
26. University of Puerto Rico Graduate School of Public Health	School/Departmental	San Juan, Puerto Rico
27. University of South Florida College of Public Health	College/Collegewide	Tampa, Florida
28. University of Texas Health Science Center at Houston School of Public Health	School/Departmental	Houston, Texas

https://journals.plos.org/plosone/article/figure?id=10.1371/journal.pone.0245892.t001

Types of DrPH programs was divided into three formats: (1) school schoolwide (college/collegewide); (2) school (college)/departmental; and (3) program/concentration. School schoolwide (college/collegewide) was used to describe a university which has a school (or college) of public health and the DrPH program is provided interdepartmentally and schoolwide (or collegewide). School (college)/departmental was used to describe a university which has a school (or college) of public health and the DrPH program was provided within its own department. Program/concentration was used to describe a university which provides a public health program with concentrations or specialties in the degree curricula. Although our study may not include all of the existing CEPH-accredited DrPH programs in the United States, a sample of 28—school/schoolwide (college/collegewide): 13, school/departmental (college/departmental): 13, and program/concentration: 2—through a collectively exhaustive approach still represents the most common DrPH programs in the United States and its territories approved by CEPH, the largest and most respected accrediting body in the field of public health.

After identification of CEPH-accredited DrPH programs, we conducted a qualitative analysis to obtain perspectives from DrPH academic leaders in each of the DrPH programs. This study was determined to be research that is exempt from IRB review under DHHS regulatory, category 2 (IRB# NCR191841). Purposive sampling was used to select participants; to reach the broadest set of perspectives possible from CEPH-accredited DrPH programs, we invited the program directors of each of the 28 selected programs to ask to participate in a semi-structured qualitative interview. We conducted in-depth interviews during February 3–25, 2020. The first author conducted all in-depth interviews, who had a wide variety of experience in conducting qualitative studies both domestically and globally. At the time of conducting interviews, he was a DrPH candidate. All of co-authors earned a doctorate degree. The first author had no established relationship with participants before the study initiated, except two participants from universities where he studied: George Washington University Milken Institute School of Public Health and Johns Hopkins Bloomberg School of Public Health. Method of approach was email invitation, with the introduction of the goal and reasons for conducting this study. The total number of completed interviews was 22, and participants were numbered in the order of interview conducted, from #1 to #22. Since it was designed as one-time 1-on-1 interview, participants were not further contacted after an interview for feedback or a repeat interview. Interviews began when the criteria of finalizing the list of CEPH-accredited DrPH program had been modified. We excluded a completed interview from Indiana University Richard M. Fairbanks School of Public Health because its DrPH program did not meet our final criteria. Thus, a total of 21 interviews fell into the final list of CEPH-accredited DrPH programs, representing 19 of the 28 programs. Almost all the interviews were conducted by telephone (20/21, 90%), followed by in person (1/21, 5%) and by online Zoom audio conference call (1/21, 5%). Participants #4 and #6 and Participants #8 and #10 were from the same institutions (Table 2). The participation rate by individuals was 48% (21 of 44 people solicited) and by program was 68% (19 of 28 programs). The decline rate was 52% (23/44); 20 of them did not respond to the invitation, 2 of them initially showed interest but stopped responding when setting up a schedule for an interview, and 1 of them showed expression of refusal. To ensure confidentiality of participants, all interviews were only audio recorded in a private and soundproof place, and there was no one else besides the participants and the first author. Many of the institutions provide multiple DrPH degrees from different departments or concentrations, which means there are several DrPH directors in the same institution. For our study, one DrPH director was considered to represent the DrPH program in general at the institution where he/she is in because the main purpose of this research was to investigate the future direction of DrPH education in general, not based on a particular major or specialty.

**Table 2:** Participants by program type

Participants (Total: 21)	Number of participated institutions (Total: 19)	Program type
12 (Participants #1, #2, #4, #6, #7, #8, #9, #10, #16, #17, #18, #20)	10	school/schoolwide (college/collegewide)
7 (Participants #5, #12, #13, #15, #19, #21, #22)	7	school/departmental (college/departmental)
2 (Participants #11, #14)	2	program/concentration

https://journals.plos.org/plosone/article/figure?id=10.1371/journal.pone.0245892.t002

Data saturation—a criterion to discontinue data collection or analysis until it has been observed that the point of no new themes or information has been reached [24–26]—is an important methodological principle in qualitative research. Collecting sample for this study was purposeful, and sampling criterion was the theoretical saturation [27, 28]. This study concluded that the theoretical saturation point was reached after completing 22nd interview and there was no need to follow up with potential participants who did not respond to the invitation, based on the fact that no new themes or code was emerged. We asked participants permission to record the conversation before each interview; 95% of interviews (20/21) were audio-recorded. A minimum duration of 30 min is generally recommended for semi-structured, in-depth interviews [29, 30]; the actual length of the interviews ranged from 13 to 40 min, averaging 24 min. The entire interview questionnaire was developed by the first author to understand the common themes and variations of CEPH-accredited DrPH programs and desirable future direction of DrPH education in the United States. For this study, we used the following questions to understand desirable future direction of DrPH education in the United States:

1. Should the program structure be a school (college) choice, or should it be more standardized across the country?
2. What changes would you expect to see in your DrPH program in the future? How is that decided?
3. What would be fundamental, desirable future directions for DrPH degrees as a whole in the United States that you would like to see in the next 5 years?

The entire interview questionnaire is available in Supplement 1. Content analysis was conducted to underpin the study. Among 21 interviews, one interview conducted without an audio recording was excluded in the analysis because there was no prepared note taker to document participant’s comments during the interview. Thus, a total number of interviews that we used for final analysis was 20, and a verbatim transcription from each of the 20 recorded interviews was separately stored as a Microsoft Word documents (docx). We used NVivo 12 Plus for Windows (QSR International, Pty, Ltd.), and the first author coded and analyzed the data. The first author conducted the coding process through the combination of deductive and inductive approach, and the second and third authors cross-checked this process to prevent any potential bias. For this study, themes and their supporting quotes—particularly related to the future directions of DrPH program in the United States—that were identified or derived from the analytical process were organized in the results section.

## Results

After the completion of qualitative data analysis, we created two categories: 1) future development of DrPH program and 2) desired future direction of DrPH program as a whole nationwide. Table 3 demonstrates those two categories and their subcategories.

**Table 3:** Professional opinions from DrPH directors

Categories	Sub-categories
Future Development of DrPH Program	Partnership and collaboration; program restructuring; curriculum restructuring; robust funding support; and further growing a student group
Desired Future Directions of DrPH Programs as a Whole Nationwide	Standardization of the DrPH program across the nation; leadership focus; recognition of the DrPH program; establishment of partnership and funding; methodology focus; application, practice and interventions; open to any career pathway

### Future development of DrPH program

Participants were asked what changes they expect to see in their DrPH programs in the future. Their answers leaned towards the DrPH program in general from their institution, not related to specialty (e.g., biostatistics or epidemiology) focus.

#### Partnership and collaboration

Eight participants emphasized the importance of establishing a partnership with local communities and universities to connect their students to various organizations. In 2019, the DrPH Coalition—a group of DrPH students and alumni across the country—was created to expand the DrPH community and support DrPH programs [31]. The academic community expected that students would lead this collaboration, and it would have a positive effect on complex public health issues around the globe:

*Our DrPH students are leaders in the DrPH community across the country and across the world. You may be aware that a group of DrPH students has gotten together and created the DrPH Coalition. They had a recent APHA meeting. They had their first conference in Philadelphia, and we also have a group of DrPH students from Boston. (Participant #2)*

#### Program restructuring

Twelve participants shared plans for restructuring their DrPH programs. Two participants are considering or have already been transitioning from departmental to schoolwide programs. Of these participants, six of them (6/12) were considering providing an online-hybrid DrPH program to accommodate students’ professional lives. Aligning with this new mode of delivery, three participants (3/12) were considering transitioning their programs into executive programs.

#### Curriculum restructuring

All of the DrPH curriculums have been restructured with various components: a better reflection of CEPH competencies; addressing the real public health issues in the twenty-first century; making a clear distinction from Master of Public Health (MPH) and PhD programs; teaching more research-related skills; providing more diverse coursework; and developing a leadership course, practicum, and qualifying examination for moving onto all but dissertation stage; and providing better guidance for dissertation development.

#### Robust funding support

All respondents indicated there was a need to secure internal and external funding to support students financially. Two participants revealed their desire to have more funds to attract competitive students and increase the diversity of the student group:

*The next big step is to try to get more funds so that we can support our students and increase diversity. . . . The number one priority right now for all of us is increasing funding for the DrPH. (Participant #6)*

#### Further growing a student group

Program directors indicated that they planned to further grow a DrPH student group by recruiting more applicants; ensuring a diverse student group, including part-time students; actively absorbing student feedback; and retaining motivated students and guiding them well:

*I’m very interested in making sure our students feel ready to be leaders but also advance in their work or move on to something different. [We] want to make sure that we've prepared them well. (Participant #15)*

### Desired future directions of DrPH programs as a whole nationwide

DrPH programs focus on educating students to become either generalists or specialists. Although branding of DrPH programs is generally important, different directions from individual DrPH programs may confuse students, faculty, and future employers.

#### Standardization of the DrPH program across the nation

Overall, none of the participants wanted to see standardization of DrPH programs across the United States. All of them advocated for the current trajectory, in which direction of structure depends on each individual program. As to how the direction of the DrPH program should be established, comments from participants were separated into two main groups—schoolwide and departmental—to analyze if there was any trade-off between them. Another factor was that it may not necessarily matter whether the direction is schoolwide or departmental if the program’s focus is health policy and management because a DrPH degree, in general, emphasizes leadership and management. Even if the degree is an executive program focusing on health policy and management, operationalized by the department, the characteristic of the degree can be functionally seen as schoolwide.

Generally, participants from schoolwide programs agreed that the core characteristic of the DrPH program, interdisciplinarity, is maintained under the schoolwide approach, when students can explore the broader concept of public health across departments. Furthermore, they highlighted that a DrPH program is a practice-based degree with a broader area of specialty and expertise than other kinds of advanced programs, and it needs to be differentiated from a PhD degree for students who pursue intensive research skills:

*We chose to make ours more of a generalist degree. . . . [Our] students probably have a broader focus across multiple domains as opposed to an intense research interest in just one very specific topic. I think that is the nature of the DrPH because of its practice-based focus. There’s more interest in theory leading to practice, and they are able to go into a broader range of work areas. (Participant #11)*

*Our students have at their disposal the resources from all departments. How that works is a little different than the way it looks on paper because sometimes students gravitate toward the faculty they interact with most, and there are some departments that are much more interactive with our students. . . . We presumably have access to all of those departments and what they're able to offer. (Participant #17)*

Likewise, participants from the departmental program would prefer to maintain specialties or emphasize that their departmental DrPH program must provide focus for students. One of the biggest benefits for DrPH students focusing on a specialty is that they can dramatically increase the possibility of becoming involved in a federal grant program. Participant #6 from a schoolwide DrPH program expressed concern about the lack of funding opportunities for students:

*Especially because the DrPH is a professional degree, not an academic degree, we do not have access to grant money to support the students like PhD students have.*

In addition, Participant #13 pointed out that developing a schoolwide DrPH program would cost a lot and would require more administration:

*We maintain the DrPH programs because everybody felt that there was a real need and purpose for them at this level of doctoral education. . . . Schoolwide program is more involved in administration [and is for] students who have been in business school or law school, who are looking at policy or a much more generic program. But we recognize the very high cost of developing a schoolwide DrPH program. (Participant #13)*

*I have a very strong bias because I work with a program that’s focused on [specialty]. . . . We look at the policy and the infrastructure within the United States for [specialty] program, unlike other programs. I wouldn’t want to lose that emphasis by creating a collegewide DrPH program. Being able to have a DrPH in [specialty] really capitalizes on centuries of [specialty] work that has led to Title [number] programs, Title [number] programs, and a national and state structure that supports [number] programming. (Participant #15)*

Some participants admitted that they had not deeply thought about standardization or said it depended on each school and its organizational structures:

*I don’t know that I have a real strong opinion about that. I haven’t really thought about that too much, but I will tell you that the idea of offering a generalist degree has been discussed, and it’s something we’re thinking about. (Participant #14)*

*I would think that it would be up to the school. Different schools have different organizational structures, departments, and things of that nature. For some academic institutions, it may be appropriate to have them housed in a department, while for others, it may make sense for them to be collegewide. (Participant #16)*

A participant from a departmental-based executive program in Health Policy and Management introduced the dynamic between schoolwide and departmental programs. Faculty from Health Policy and Management started a schoolwide DrPH program but eventually switched to housing it in their department because other departments did not fully appreciate or clearly understand the concept of a schoolwide leadership-focused DrPH degree. Instead, the other departments continued to treat it as another PhD program. It appears that as long as the main focus of leadership and management remains intact, the program structure—schoolwide or departmental—could simply be an administrative issue:

*We may eventually look at a schoolwide DrPH. . . . I think that would be in addition to [our departmental DrPH program]. We had a schoolwide DrPH when we first started. . . . . We found that other departments didn’t have an understanding of what this program was about, and they just treated it like a PhD program. So we moved it into the department because our department was the one who embraced it and understood the concept of a leadership-focused degree as well as a practice-based degree. (Participant #19)*

#### Leadership focus

Four participants highlighted that DrPH programs should focus on leadership. A DrPH program does not necessarily steer students into an academic route; rather, it should steer them into a leadership path in which they are incorporated into large organizations. DrPH programs should be recognized for preparing public health leaders by teaching them solid leadership skills, analytical thinking skills, and advanced applied skills:

*It’s all [about a] leadership degree. The Doctor of Public Health should be seen as an individual . . . [as a] leader and a subject matter expert who has been trained in leadership skills and the analytical thinking skills. (Participant #1)*

*I think paying greater attention to the need for this kind of leadership and building with these kinds of applied skills . . . is really different from an academic background. Our students do not go down an academic route but go into leadership roles at large organizations. That’s what I hope the DrPH thing will receive greater recognition for in the field. (Participant #13)*

#### Recognition of the DrPH program

Almost three-quarters of the participants were eager to see DrPH programs get the recognition they deserve (14/20). Because recognition of the DrPH degree is crucial to its continued existence, most of the comments from 14 participants were briefly quoted in this section.

Two of these 14 participants wanted to see the DrPH degree as a premier practice degree. Participant #20 mentioned that just as medical schools offer PhD degrees even though their main purpose is to train MDs, the DrPH degree should continue to maintain the integrity of the profession and also to add diverse strengths to the workforce by providing different types of degrees—both academic and professional degrees—in the field of public health:

*Let [the DrPH] be recognized as the premier practice degree in public health. (Participant #2)*

*There are a lot of med schools in the country. . . . They may have PhDs but they primarily trained practitioners—same with nursing, same with pharmacology, same with dentistry, right? If we consider ourselves a health science professional, why in the world would we give up a professional degree? . . . We thought it was important to maintain the integrity of the profession by keeping our DrPH. (Participant #20)*

Four of the participants (4/14) felt strongly that the DrPH degree should be more respected and recognized as a legitimate doctoral degree, rather than being mistaken for a less rigorous PhD. A DrPH program is different from a PhD program that can bring uniqueness to academia and the community. It is also important to change people’s misunderstandings regarding the difference between a DrPH and a PhD:

*I think that some people view a DrPH as lesser than a PhD, and I don’t really think that’s the case. I think they’re different. I’d like to see the degree evolve in such a way that it’s truly recognized for the benefits and expertise it brings to the public health practice community. (Participant #11)*

*I would like to decrease this sort of false dichotomy saying that a PhD is a more rigorous degree than a DrPH. I think they really are trying to create different types of skills, but one shouldn’t be seen as lesser than the other. (Participant #13)*

*I don’t want to see [that] people [are] thinking the DrPH is just an easy PhD. It’s not, and I want to make sure that it maintains the respect and the integrity that it deserves. (Participant #20)*

Five participants (5/14) wanted the DrPH degree to be distinct and separate from the MPH degree and/or PhD degree. Although it has been 100 years since a DrPH degree was introduced in the United States, there is still active debate on the question, “What is the difference between a DrPH and a PhD?” There should be a much stronger boundary between the DrPH and the PhD along with a clearer definition of the DrPH, specifically addressing what public health professionals with this degree actually do in the real world, what benefits they offer, and what services they provide. Regarding this issue there were several concerns about CEPH competencies among the participants. Participant #18 thought CEPH competencies do not actually reflect what the DrPH program must teach (e.g., not a strategic plan, but a strategic management of practice and application in the community):

*Additional [CEPH] concentrations [need to] make sure that it ([DrPH degree]) is very distinct from the MPH and from PhD programs and the dissertation. . . . I would like, in the future, to see more programs make that very clear for students because I feel like most of the time, what I do is explain the difference between the PhD and the DrPH. (Participant #10)*

*I’d like there to be a much stronger boundary around the definition of what a DrPH is and does in the world. . . . We should all work to our strengths, but I think there’s not a lot of clarity around what a professional doctorate is and what the recipient does in the field. (Participant #17)*

*The CEPH competencies say you have to know how to do a strategic plan. Well, we teach strategic management. It’s not about the plan, because most plans sit on the shelf. It’s about how you lead an organization strategically in an ongoing way, and I would love for us to have more dialogue around that distinction. (Participant #18)*

Last, three participants (3/14) looked forward to seeing more DrPH alumni taking the lead in decision-making and policymaking by serving in high-ranking positions at top health-related governmental and nongovernmental organizations. They also hoped that people who are in senior positions in those organizations could receive public health training, even if they already hold a medical or a nursing degree:

*I had previously worked in our county, and I would like to see more DrPHs in roles that normally go to MDs. Because I noticed when I was working in our health department, there’s a lot of masters-level folks, but when you get to the more senior positions, it’s all MDs. I think it would be good if we started to see more DrPHs integrated into those role [s]. (Participant #8)*

*I would like to see individuals who have DrPHs have a seat at the table for different advisory groups like National Institutes of Health, health resources, and services administration. They need a seat at those tables to be part of the decision making, whether it’s for policy, practice, or research. (Participant #15)*

*I would like to see the executives at the top health-related organizations, the decision-makers, have DrPH degrees. I would like to see more physicians and nurses with public health training. (Participant #20)*

#### Establishment of partnership and funding

Two participants highlighted that funding opportunities and partnerships with various organizations should be more established for students, to ensure equal opportunities for all:

*We have to keep an eye on cost to make sure that we find financial opportunities, so that the doors are open to all students who potentially want to obtain a DrPH, I think right now there isn’t equity. (Participant #7)*

*A lot of attention to collaborative effort, to be thought leaders, to look at trends and analyze them, to look at that broader scope of systems and partnerships across governments, NGOs, and donors. (Participant #13)*

#### Methodology focus

Although all 20 participants mentioned throughout the interviews that the DrPH is a professional degree, two highlighted that DrPH programs should still focus on methodology to train students in the research skills that translate into public health practice. It is notable that this professional opinion was from participants whose academic backgrounds were PhDs:

*I think maybe this is my personal biased because I consider myself more of a quantitative researcher, and able to see more emphasis placed on quantitative methods and translating research into public health practice. I think that is something that DrPH programs across the nation should focus on and pay more attention to. (Participant #16)*

*I think moving in this trajectory is a good path, and I think [we should continue] to make sure the DrPH students still have strong methodological skills. (Participant #21)*

#### Application, practice, and interventions

Six participants (6/20) emphasized application, practice, and interventions through case-based learning. They felt that first and foremost, a DrPH program should address emerging, complex public health issues. Case-based learning would provide DrPH students with insight into working on real public health issues across the globe, such as controlling infectious diseases, global warming, and the social determinants of health. DrPH programs should train and inspire students to develop the leadership skills needed to be positioned for success in addressing large, complex public health issues and to move up the ladder into higher-level leadership roles in key organizations:

*We have to look at what challenges the 21st century in public health is posing, such as infectious diseases or any other public health problems affected by the social determinants of health. I think that in the future, the best programs in the country will be those who actually address more cases. (Participant #4)*

*DrPH graduates have to play an increasing role in solving some of the most important problems in society, which are obviously public-health–related but not necessarily labeled as public health problems. . . . I really think the DrPH programs in the country as a whole have to keep in mind that what we’re training is leaders. . . . We’re going to have to go one level higher and actually train people to address big, complex problems. (Participant #6)*

One participant indicated that DrPH programs must align with the needs of the public health workforce to successfully address complex changes in public health. As such, DrPH programs should consider all available resources to find a niche in the field of public health for students:

*We have to continue to keep an eye on that, to make sure schools of public health and in particular DrPH programs are really aligning with the needs of the public health workforce. . . . We need to keep working on finding resources, so that we can promote equity across the workforce for all these students to have an equal opportunity to achieve their academic pursuits through DrPH programs. (Participant #7)*

One participant mentioned the value of creating a shared vision across the DrPH programs of what this degree can provide for students:

*I think we should really create a shared vision around what the DrPH really provides for our discipline. For me, the root of it is our practitioners; not everybody’s going back into academia. (Participant #18)*

#### Open to any career pathway

Three participants said it was important for us to respect and be open to various types of DrPH programs to offer the opportunity for students to self-select a pathway, either academia or practice, that would work best for them. Even if the DrPH program was originally meant to prepare public health professionals who are expected to implement evidence-based interventions in the field, it is still valuable to ensure the diversity and variations of DrPH programs, including research-focused ones, throughout the university and college system in the United States:

*DrPH programs, in general, have made huge strides in terms of getting students to where they should be and preparing them to go into practice or academia. (Participant #10)*

*What’s great about the United States is its great educational system. The university and college system [in the United States] has excellent neurological programs. I think the diversity of programs is valuable. I wouldn’t want to see every university offer exactly the same kind of doctoral program. I think that’s important, because students have different interests. (Participant #14)*

Participant #17’s comment clearly describes how DrPH programs, in general, look across the nation:

*We should reach some consensus on this point, because as my colleague always says, “If you’ve seen one DrPH program, you’ve seen one DrPH program.” We're all very different, and it would be great if we have variation and we also did a common thing in different ways, so that some students could self-select into what works for them. (Participant #17)*

## Discussion

Over the past decade, DrPH programs have been discontinued or created in universities across the United States [21, 22, 32] presumably due to a lack of common understanding of intention and goal of DrPH education. Participants did not seem to favor the concept of a common curriculum across the country because they were preoccupied with managing their own DrPH program. However, if there were a more consistent format for a DrPH degree, then universities would not have any difficulties in designing a DrPH curriculum and differentiating it from a PhD curriculum, which would ultimately improve DrPH education for correctly educating and fostering the future generation of public health researcher-practitioners. In addition, establishing generally accepted U.S. DrPH structures and curriculum models would also provide a good example for other countries that wish to launch the DrPH degree and do benchmarking and strategic planning. Thus, suggesting a desired future direction for DrPH programs in the United States through this study must be beneficial to institutions in locations that plan to further strengthen or newly establish such a degree.

A key strength of the study was that it represented a comprehensive examination of the future direction of DrPH programs by conducting in-depth interviews with DrPH directors from active CEPH-accredited DrPH programs in the United States. Given the fact that there have been few collective efforts of professional opinions from DrPH directors to have a better understanding of desired future directions of DrPH education, the findings from this study will add to the rapidly expanding field of public health practice that well-qualified DrPH alumni are expected to carry out.

This study had a few limitations. First, although understanding why some DrPH programs recently have stopped accepting students was not the main purpose of the study, a lack of interviews with directors from those discontinued programs might have limited the scope of the qualitative research. For example, one of the discontinued DrPH programs, the University of Pittsburgh Graduate School of Public Health, was included in the initial list of active CEPH-accredited DrPH programs without realizing that they have already suspended a DrPH program. Although an interview invitation and a reminder were sent to a DrPH director who was introduced in the University of Pittsburgh Graduate School of Public Health webpage, there was no reply to the invitation. If the study had expanded the target interviewees to former directors from DrPH programs discontinued in the last 10 years, other insights or thoughts about the future of the DrPH program across the country might have been collected.

Second, this study has only examined the opinions from faculty who are in leadership roles at DrPH programs at educational institutions. Having the other perspective, that of the CEPH staff, DrPH applicants, current students, and alumni, would have enriched the variety of the qualitative results. CEPH would have a clearer concept of the distinction between DrPH and PhD programs and could create a roadmap for transforming a DrPH degree into a unique, professional terminal degree in the field of public health. Likewise, it would have been helpful to have opinions from DrPH applicants to ask their expectations of the DrPH program and the reasons they chose a DrPH degree over a PhD degree. Current DrPH students and DrPH alumni could state their views of DrPH programs by sharing their experiences and thoughts, such as what kind of gap they felt between their initial expectations when they began and the kind of DrPH education they received, and, furthermore, what type of DrPH curriculum they are eager to see for future DrPH students.

Future studies could include other perspectives such as CEPH staff, DrPH applicants, current students, and alumni, to enrich the variety of the qualitative results. Including a wider range of participants would result in providing a clearer distinction of a DrPH degree from MPH or PhD degree and could create a roadmap for transforming the DrPH degree into a unique, professional terminal degree in the field of public health.

## Conclusions

The DrPH program director interviewees did not favor standardization of curricula across the country but preferred to respect the unique direction that each of the DrPH programs pursues for accommodating students’ various interests and future career pathways. Each was eager to continue developing his/her program for educating and empowering future leaders of public health through different program structures, specialties, and concentrations. In addition, interviewers suggested a desire for common understanding of the DrPH degree as distinct from an MPH and a PhD. To meet the expectations of DrPH directors who are core leaders for designing and developing DrPH programs, the DrPH degree should emphasize a holistic approach, from research methods training, to integrated teaching and learning, to interprofessional training and leadership [33, 34].

It is suggested that all DrPH programs should support students both academically and practically, so the students can choose career paths without encountering any limitations. As public health researcher-practitioners, DrPH graduates should act as mediators between those two areas, serving either as faculty at educational institutions or as practitioners in leadership roles within organizations. Thus, the future of DrPH programs in the twenty-first century should aim at effective interdisciplinary public health approaches that are linked to both and applied sectors.

## Supplementary Information

**Additional file 1.**

## Data Availability

The datasets used and/or analyzed during the current study are available from the corresponding author on reasonable request.

## Abbreviations

APHA: American Public Health Association
ASPH: Association of Schools of Public Health
ASPPH: Association of Schools and Programs of Public Health
CEPH: Council on Education for Public Health
DrPH: Doctor of Public Health
