# Supplementary material for: Future directions of Doctor of Public Health education in the United States: a qualitative study
Source: BMC Public Health. 2021 Jun 3;21:1057. doi: 10.1186/s12889-021-11086-z (PMC8173852; doi:10.1186/s12889-021-11086-z)
Supplement: Supplementary file 1 — Additional file 1. [file 12889_2021_11086_MOESM1_ESM.docx]

Supplement 1. Interview questionnaire for DrPH program directors

| **Topics** | **Questions** |
| --- | --- |
| Before starting: For the purpose of transcription, may I record our conversation? | |
| 1. Strength | What are the strengths of the DrPH program at your school, and how could these strengths be used to successfully educate and empower the future leaders of public health? (If interviewee can’t answer this right away, the following prompts are recommended: school location, access to public health work, priorities, core expertise, or any other factor.) |
| 1. Weakness | What challenges do you face in the DrPH program? (If interviewee can’t answer this right away, the following prompts are recommended: financial aid, tuition costs, faculty capacity, attention to mentoring DrPH students, or any other factor.) |
| 1. Program Type, Residency Requirement, and Students | For schoolwide DrPH directors: What is the relationship between the DrPH program and the other departments? I’m wondering whether the interdisciplinary DrPH program communicates and collaborates with other departments to ensure interdepartmental doctoral-level education for DrPH students.  For departmental-based DrPH directors: I am wondering about the relationship between the DrPH program in your department and DrPH programs from other departments?  How many students are recruited annually? What is the total program size?  How do students normally find and complete the practicum? How do you decide whether the applied practice experience (practicum) differs substantially from a student’s current job description? Or do you allow students to complete the practicum from their current job?  For schools that did not provide information: What are the minimum hours or credits to complete the practicum?  Leadership, management, and governance are some of the DrPH foundational competencies in CEPH criteria. How has your DrPH program focused on its leadership course? How have leadership courses been developed?  For schools that accept both part-time and full-time students: part-time and full-time students may have different expectations and perspectives. How has your DrPH curriculum tried to satisfy all students who are in the same class?  General: How has your DrPH curriculum tried to satisfy all students in the same class who may have different expectations and perspectives?  For schools that accept both part-time and full-time students: How would you like to further develop the curriculum to embrace both part- and full-time students as well as students from various backgrounds who have different expectations and perspectives?  General: How would you like to further develop the curriculum to embrace students from various backgrounds who have different expectations and perspectives?  For schoolwide DrPH directors: Many of the DrPH programs in different schools are still departmentally based. How was your DrPH program determined to be interdepartmental? I would like to hear your opinion about schoolwide DrPH programs vs. departmental-based DrPH programs.  For departmentally based DrPH directors: Some of the departmentally based DrPH programs have or will have changed to schoolwide DrPH programs. Would your DrPH program be maintained as departmentally based? I would like to hear your opinion about schoolwide DrPH programs vs. departmentally based DrPH programs. |
| 1. Changes from past to present | A lot of DrPH programs have changed over the years and are still evolving. In what way is your program evolving in terms of structure, curriculum, mission, or vision?  During the development of your DrPH program, are there any guiding principles or values that your program has followed? |
| 1. Heading for the future | Should the program structure be a school (college) choice, or should it be more standardized across the country?  What changes would you expect to see in your DrPH program in the future? How is that decided?  Furthermore, in your opinion, what would be fundamental, desirable future directions for DrPH degrees as a whole in the United States that you would like to see in the next five years? |
| 1. Etc. | Is there anything else that you would like to share about your DrPH program? |

Adopted from Park C, Migliaccio G, Edberg M, Frehywot S, Johnson G: Analysis of CEPH-accredited DrPH programs in the United States: A mixed-methods study. PloS one. 2021;16(2):e0245892, Table 2. <https://journals.plos.org/plosone/article/figure?id=10.1371/journal.pone.0245892.t002>
